# Carbon nanomaterials as smart interfaces in ultrathin films for high-performance electrochemical sensors: a critical review

**DOI:** 10.1039/d5ra04565g

**Published:** 2025-08-15

**Authors:** Monika Poonia, Amar Singh

**Affiliations:** a Department of Civil and Environmental Engineering, Wayne State University Detroit MI USA monika.poonia@wayne.edu; b Computational Biology Program, The University of Kansas Lawrence KS USA

## Abstract

Carbon nanomaterials (CNMs) have emerged as pivotal components in the evolution of electrochemical sensors (ECSs), owing to their exceptional electrical conductivity, biocompatibility, and versatility across various analytical applications. Recent advancements in nanoengineered thin films of CNMs have yielded robust electrochemical sensing systems with enhanced analytical capabilities. This review article offers a detailed analysis of the use of nano-engineered CNMs thin films, with a particular focus on layer-by-layer assembly, Langmuir–Blodgett, and Langmuir–Schaefer deposition techniques for the development of high-performance ECSs. By presenting a holistic overview of the field, this article explores the diverse CNMs employed in ECS development, examining their synthesis and the ongoing quest to optimize material properties for enhanced sensor performance and technological advancement. It highlights the remarkable electrochemical properties of CNMs and their significant potential in the sensitive and selective detection of a wide array of substances, including chemicals, pharmaceutical compounds, biological analytes, environmental pollutants, and gases. This review summarizes the current status and future potential of nano-engineered CNM thin films in electrochemical sensing applications. Additionally, it highlights the need for further research to address challenges related to thin film stability, reproducibility, and material selectivity for sensing applications at the intersection of nanotechnology and electrochemical sensing.

## Abstract 2

Carbon nanomaterials (CNMs) as electrochemical sensors (ECSs).

## Graphical abstract

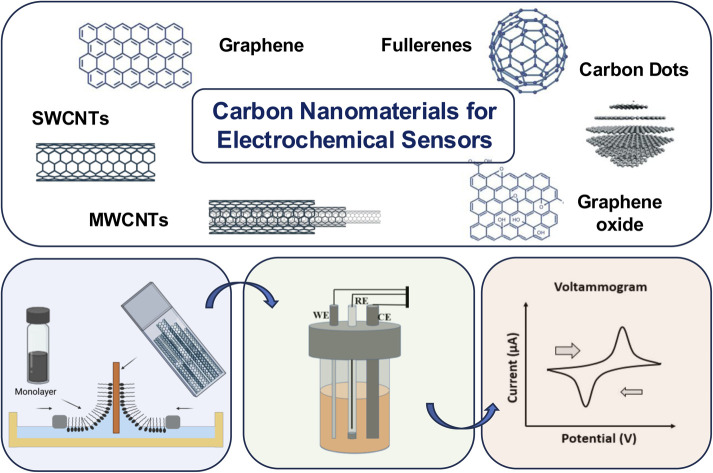

## 1. Introduction

The rapid advancement of sensing technologies has underscored the importance of developing highly sensitive and selective sensors for a wide range of applications, including environmental monitoring, healthcare diagnostics, and industrial process control. Among various sensor platforms, electrochemical sensors (ECSs) have attracted significant attention due to their inherent advantages such as high sensitivity, low cost, rapid response, and ease of miniaturization.^1^ Carbon nanomaterials (CNMs) have emerged as powerful components in developing high-performance ECSs, particularly when integrated into ultrathin films. These materials, such as graphene, fullerene, carbon nanotubes (CNTs), and carbon quantum dots (CQDs), possess large surface-to-volume ratios and high electric conductivity, making them highly effective and well-suited for applications in ECSs.^2–6^

Electrochemical analysis provides several advantages over alternative detection methods such as luminescence, chromatography, and spectroscopy, including high sensitivity, selectivity, accuracy, low cost, reliability, and ease of use.^7,8^ Various analytical techniques, including linear sweep voltammetry (LSV), differential pulse voltammetry (DPV), cyclic voltammetry (CV), and chronoamperometry are utilized to investigate electrochemistry of electroactive species in solution.^2,9,10^ Electroanalytical methods rely on the physical and chemical properties of electrode surfaces, the influence of applied potential, adsorption phenomena, and coatings used on the electrode surface to enhance detection. In recent years, nanostructured thin films of CNMs have captured significant attention as advanced electrode materials in ECSs, owing to their tunable thickness, porosity, and surface functionalization, which enable enhanced selectivity, sensitivity, and stability in sensing performance.^11–15^ The nanostructured electrodes are highly promising because they enhance the exposure of active sites and improve ion transport rates. For instance, an open two-dimensional (2D) nanostructure that contains adsorption sites can shorten the pathways for both electrons and ions. The gap between the sheets of 2D material serves as an effective channel for ion transport, while the integrated adsorption sites on sheets may enhance electronic conductivity.^16^ Several methods such as spin coating, vacuum filtration, spray coating, and chemical vapor deposition (CVD) have been employed to fabricate ultrathin films of CNMs. These techniques enable tunable film properties for diverse applications, though often at the cost of precision or molecular alignment achieved by more controlled assembly methods.^17^ A sophisticated approach for controlling the arrangement of CNMs in a 2D plane is through the Langmuir–Blodgett (LB) and Langmuir–Schaefer (LS) methods. These techniques are effective in creating supramolecular assemblies with a precisely controlled layered structure by transferring a nanomaterial monolayer formed at the air–liquid interface.^18–20^ Controlled organization and densification of materials promote the formation of ion-conductive channels and porous architectures, which are highly beneficial for electrochemical sensing platforms. Such structured arrangements facilitate efficient ion transport and create well-ordered, conductive, and biocompatible films. These features enhance electron transfer kinetics and analyte recognition, both of which are essential for achieving high sensor performance. Additionally, the layer-by-layer (LbL) characteristics of LB films enables the fabrication of both two and three-dimensional (3D) heterostructures with various materials that are held together by non-covalent interactions.^21–23^ Several studies have demonstrated the use of LbL and LB assembly to fabricate metal-oxide or metal–organic framework (MOF) thin films for high-performance electrochemical and chemiresistive sensing.^24,25^ Notably, Yao *et al.*^26^ employed a spray-LbL liquid-phase epitaxial method to produce highly oriented, thickness-controlled conductive MOF (Cu_3_(HHTP)_2_) nanofilms, achieving exceptional room-temperature NH_3_ sensing performance with nanometer resolution per cycle (≈2 nm growth per cycle), high sensitivity and fast response.

This review presents a comprehensive analysis of recent advancements in CNM-based ultrathin films for high-performance ECSs. It systematically discusses the diverse carbon nanostructures employed in thin-film sensor platforms, along with their synthesis methods, film fabrication techniques; including LB deposition, LS deposition, and LbL deposition; and the fundamental mechanisms underlying their enhanced electrochemical performance. Additionally, the review covers the electrochemical sensing applications of LB films of CNMs, including gas sensing, biosensing, and environmental sensing. This review highlights recent breakthroughs in sensor performance and discusses how carbon nanostructures have improved detection limits, expanded the range of detectable analytes, and enabled multi-analyte sensing platforms. Furthermore, it identifies key challenges and opportunities in the field, including strategies to enhance long-term stability, selectivity, and scalability for commercial deployment. By providing a comprehensive overview of thin film carbon nanostructures in electrochemical sensing, this review aims to guide future research directions and enhance the advancement of next generation electrochemical sensing technologies.

## 2. Carbon based nanomaterials

CNMs, such as CNTs, CQDs, graphene, graphene oxide (GO), and fullerenes have emerged as promising materials for ECSs.^27^ Carbon atoms have a valency of four and can form single, double, and triple covalent bonds with each other or with other elements. They can create long chain of atoms, which demonstrates polymerization phenomenon. Due to their electronic structure and atomic size, carbon atoms can adopt various physical structures with unique properties, even though they share the same chemical composition. This versatility arises from their ability to undergo sp, sp^2^, and sp^3^ hybridizations, facilitated by the small energy gap between 2s and 2p electron orbitals. Two prominent allotropes of carbon are diamond, featuring sp^3^ hybridization, and graphite, exhibiting sp^2^ hybridization.^28^ CNMs have numerous technical applications across various fields, such as energy storage,^27^ environmental technology,^29^ and biomedical diagnostics,^30^ due to their low toxicity and capability for large-scale production.^31,32^

**Fig. 1:**
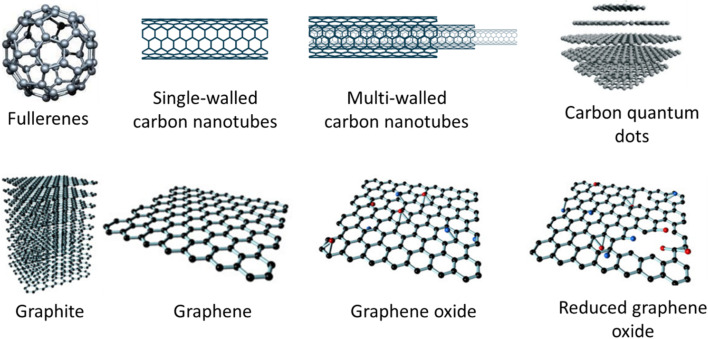
Schematic illustration of individual allotropes of carbon nanomaterials (CNMs).

The geometric arrangement of particles in nanomaterials serves as a fundamental criterion for their categorization. Key allotropic forms of nanocarbon include 0D nanodiamonds, 1D nanotubes, and 2D graphene nanosheets, which can serve as foundational building blocks for nanocomposites.^33^ The most common types of CNMs are shown in Fig. 1, and their synthesis processes are discussed further. The synthesis procedures described for CNMs represent standard approaches intended to produce high-quality CNMs with broad applicability across various research and application areas, including but not limited to electrochemical sensing.

### 2.1. Fullerenes

Fullerenes are an allotrope of carbon, often described as molecular carbon or carbon molecules.^34^ The fullerene family encompasses a range of carbon-based clusters (C_*n*_, where *n* > 20), characterized by carbon atoms arranged on a closed, spherical surface. These atoms are typically positioned at the vertices of a combination of pentagonal and hexagonal rings. In fullerenes, the carbon atoms predominantly exhibit sp^2^ hybridization and are interconnected through covalent bonds. Among the various members, C_60_ is the most extensively studied and well-characterized. This molecule possesses a highly symmetric structure composed of 60 carbon atoms forming a truncated icosahedron, consisting of twelve pentagons and twenty hexagons. The diameter of C_60_ is approximately 0.7 nm. They have unique electrochemical properties and have been used as mediators in electrochemical biosensors to enhance the rate of electron transfer.^35,36^

#### 2.1.1 Synthesis

Fullerenes, such as C_60_, are typically synthesized using methods such as laser ablation and arc discharge.^37^ An alternative and rational synthetic strategy involves the use of specifically engineered polycyclic aromatic hydrocarbon (PAH) precursor molecules that incorporate the requisite carbon framework for constructing the desired fullerene cage. These precursors can be transformed into the target fullerene structure through a tandem intramolecular aryl–aryl coupling reaction, effectively ‘zipping’ the framework into a closed cage. The arc-discharge method is a widely used technique for producing gram-scale quantities of fullerenes in laboratory settings. This process involves generating an electric arc between two graphite electrodes in an inert helium atmosphere. The resulting carbonaceous soot contains approximately 10–15% soluble fullerenes, which are subsequently extracted and purified.^38,39^

### 2.2. Carbon nanotubes

CNTs, first discovered by Sumio Iijima in 1991, represent a distinctive allotrope of carbon.^40^ These nanotubes are composed of a single layer of graphene rolled into a seamless, hollow cylinder. CNTs can be categorized into single-walled carbon nanotubes (SWCNTs) and multi-walled carbon nanotubes (MWCNTs) (Fig. 1). SWCNTs typically have diameters ranging from 0.4 to 2 nm and exhibit either metallic or semiconducting properties, depending on their chirality and diameter. In contrast, MWCNTs consist of multiple concentric layers of graphene sheets, with diameters ranging from 1 to 10 nm, depending on the number of graphene layers. The exceptional structural and electronic properties of CNTs make them highly suitable for a variety of applications, including in micro- and nanoelectronics, gas storage, conductive polymers, composites, and biosensing technologies.^33^

#### 2.2.1 Synthesis

CNTs can be synthesized through several techniques, including arc discharge, laser ablation, and CVD.^41^ The CVD method entails the decomposition of hydrocarbons over a transition metal catalyst, resulting in the growth of SWCNTs, MWCNTs, and coiled CNTs. Both arc discharge and laser ablation methods have also been employed for CNTs production. The arc discharge method involves generating an electric arc between two graphite electrodes in a helium atmosphere, while laser ablation uses a high-power laser to vaporize a graphite target within a high-temperature furnace. These synthesis techniques have played a pivotal role in the large-scale production of CNTs and have facilitated extensive research into their properties and potential applications across various fields.^42,43^

### 2.3. Graphene and its derivatives

Graphene, discovered by Andre Geim and Konstantin Novoselov in 2004, earned them the Nobel Prize in Physics in 2010.^44^ Graphene is a 2D carbon allotrope consisting of a single layer of carbon atoms arranged in a hexagonal lattice. The carbon atoms in graphene exhibit sp^2^ hybridization, connected by robust σ- and π-bonds, forming a 2D hexagonal crystal structure with a carbon–carbon bond distance of 0.142 nm. As the fundamental building block of various carbon allotropes, graphene can be transformed into 0D fullerenes, rolled into 1D carbon nanotubes, or layered to create 3D graphite (as shown in Fig. 1). Its 2D structure provides an exceptionally high surface area, making it highly suitable for a variety of applications. Graphene is a semiconductor with a zero bandgap, which renders its electronic properties highly sensitive to both electron-donating and electron-withdrawing molecules. Furthermore, graphene demonstrates outstanding mechanical strength, thermal conductivity, optical transparency, and flexibility. These combined physical, chemical, and electrical properties position graphene as a promising material for the development of high-sensitivity, label-free ECSs.^5,35,45^ GO on the other hand, is an oxidized form of graphene, fabricated by the oxidation of graphite. It is a single-atomic-layered material that is dispersible in water and other solvents. GO is non-conductive due to the oxygen-containing groups in its lattice. However, it can be reduced to graphene through chemical, thermal, or electrochemical processes, resulting in reduced graphene oxide (rGO). The reduction process is vital as it has a large impact on the quality of the rGO produced. GO and rGO have various applications in electronic, optics, chemistry, energy storage, and biology.^46^

#### 2.3.1 Synthesis

In recent years, various techniques have been developed for synthesizing graphene. The most commonly employed methods include chemical exfoliation, chemical synthesis, thermal CVD, and mechanical exfoliation.^47^ Other reported techniques encompass nanotube unzipping and microwave-assisted synthesis. Mechanical exfoliation using an atomic force microscope (AFM) cantilever has been shown to produce few-layer graphene, although the process is limited by the thickness of the graphene, which typically ranges around 10 nm, comparable to 30-layer graphene. The chemical exfoliation method involves the insertion of large alkali ions between graphite layers to facilitate exfoliation in solution. Chemical synthesis, a related process, involves the preparation of graphite oxide, its dispersion in a solution, and subsequent reduction with hydrazine. Among these, catalytic thermal CVD remains the most prominent technique for large-scale graphene production.^47–50^

### 2.4. Carbon quantum dots

In 2004, Xu and colleagues inadvertently discovered CQDs while working on the separation and purification of SWCNTs.^51^ CQDs are small carbon nanoparticles, typically less than 10 nm in size, that exhibit unique properties and have found widespread applications across different fields. These nanoparticles are particularly noted for their relatively strong fluorescence and display electronic and optical characteristics similar to those of quantum dots. Additionally, CQDs offer low toxicity, excellent stability, and biocompatibility, making them suitable for use in ECS applications. Owing to their tunable photoelectric properties and high surface area, CQDs have been utilized in a wide range of applications, such as bioimaging,^52^ biosensing, electrochemical biosensing,^53^ drug delivery, photodynamic therapy in cancer treatment, and pharmaceutical formulations.^54,55^

#### 2.4.1 Synthesis

CQDs can be synthesized using various methods, including microwave heating, hydrothermal synthesis, and carbonization.^56^ These methods allow for the control of CQD size and properties. In microwave assisted methods, CQDs can be produced *via* the polymerization of molecular precursors, such as glucose, sucrose, and citric acid.^57^ CQDs can also be synthesized through a single-step pyrolysis process using branched polyethyleneimine (PEI) and citric acid at temperatures exceeding 200 °C under vapor pressure.^57–61^

### 2.5. Other carbon nanomaterials

Beyond CNTs, graphene, fullerenes, and CQDs, several other carbon-based materials have been identified, including carbon nanohorns, graphene quantum dots, nanodiamonds, and carbon nanofibers.^62–64^ These materials exhibit diverse structures and properties, offering vast potential for applications in areas such as electronics, energy, and biotechnology. Ongoing research continues to explore their synthesis and properties, with these CNMs showing considerable promise for future technological innovations.^65^ Interestingly, boron-doped diamond (BDD) electrodes are increasingly favored in electrochemical sensing due to their wide potential window, minimal background currents, and resistance to fouling, making them ideal for detecting biomolecules, environmental toxins, and redox-active species.^66^ BDD films grown *via* surface functionalization using LbL assembly, such as alternating Au nanoparticles and polyelectrolytes, has been shown to enhance bioelectrocatalytic performance in microbial systems.^67^

## 3. Langmuir–Blodgett and Langmuir–Schaefer methodology

Ultrathin films can be described as mono- or multilayers of nanomaterials and nanostructures that can be created by controlling the properties of individual building blocks/components and how these are assembled. The LB and LS techniques offer precise control, high homogeneity, versatility, and the ability to engineer well-organized thin films of CNMs, making it a powerful tool for the fabrication of ultrathin films with desired properties and functionalities.^19,68–72^

**Fig. 2:**
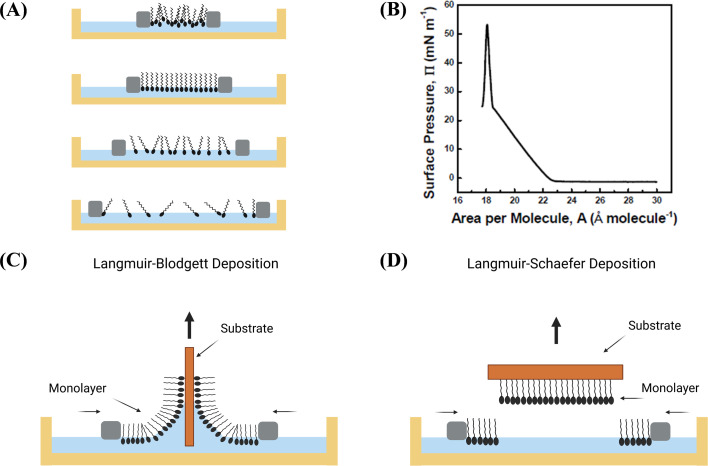
Schematic illustration of (A) Langmuir monolayer formation at air/water interface (commonly using water as subphase) with amphiphilic molecules upon barrier compression. (B) Different phases of the monolayer depicted in surface pressure–area isotherm. Schematic illustration of basic principles of transfer of monolayer from liquid surface to substrate through (C) LB deposition (vertical dipping) and (D) LS deposition (horizontal dipping) method.

The LB method involves LbL molecular-scale assembly, where ordered domains formed at the air–water interface are directly transferred onto a target substrate without the need for intermediate processes. This method is typically carried out using LB troughs, where a Langmuir monolayer (LM) floating on the surface of a chosen subphase (usually water) is compressed by bringing two opposing barriers together. The LM plays a crucial role in LB and LS thin film deposition, providing a controllable platform for assembling molecules into well-defined films with unique properties. LM is a single layer of molecules, typically about one molecule thick, at the air–liquid interface of a Langmuir trough. LM compatible materials are typically amphiphilic molecules, possessing a hydrophilic head group and a hydrophobic tail group. The hydrophilic heads point down towards the water, while the hydrophobic tails stick up towards the air. These molecules must be soluble in organic nonpolar and water-immiscible solvents.^19,68–71^ The process of formation of LM at air–liquid interface involves several key steps. First, the amphiphilic molecules are dispersed in an organic solvent to form a homogeneous solution. Next, a Langmuir trough is used to spread the nanomaterial solution at air–water interface (Fig. 2A), where the nanomaterials self-assemble into a monolayer due to the balance of intermolecular forces. The monolayer is compressed using Teflon barriers to achieve desired surface pressure, leading to the organization and alignment of the nanomaterials within the monolayer.^72,73^ Measurement of the surface pressure–molecular area (π–A) isotherm is a common evaluation method for LMs (Fig. 2B). This method provides insights into the organization and packing of the nanomaterials within the monolayer.^74^

The monolayer is then transferred onto a solid substrate using the LB deposition method, which involves vertically raising or lowering the substrate through the monolayer at a controlled speed to achieve the desired film thickness (Fig. 2C). This transfer process allows for precise deposition of the monolayer onto the substrate, enabling controlled alignment and organization of the nanomaterials.^75^ By repeating the dipping and transfer process, multilayer structures with tailored properties can be built. The deposition type achieved is influenced by several factors, including characteristics of the spread film, composition and temperature of the subphase, surface pressure during deposition, deposition rate, the type and properties of the solid substrate, and the duration for substrate is stored between deposition cycles. The LB technique enables precise deposition of monolayers onto solid substrates, with the arrangement of these layers classified as X-, Y-, or Z-type depending on the deposition sequence. In X-type deposition, monolayers are transferred onto the substrate during the downstroke (immersion), while Z-type deposition involves transfer during the upstroke (emersion). The most common configuration is the Y-type, where monolayers are deposited during both immersion and emersion, resulting in a head-to-head and tail-to-tail orientation of the molecules. These deposition types allow for the fabrication of multilayer structures with controlled thickness and molecular organization, which is critical for tailoring the properties of thin films for sensing applications.

In contrast to the LB technique, the LS deposition method involves transferring monolayers onto a solid substrate by horizontally moving the substrate through monolayer at a controlled speed, allowing for the precise control of film thickness (Fig. 2D).^19,76^ LS deposition is a variation of the LB deposition method, offering unique advantages in certain cases. The substrate is carefully oriented horizontally and brought into contact with the monolayer. The monolayer transfers onto the substrate, forming a uniform, single-layer film. The substrate is slowly and smoothly lifted back up, ensuring the monolayer remains intact. LS often works better for rough or non-planar substrates.^77^

**Fig. 3:**
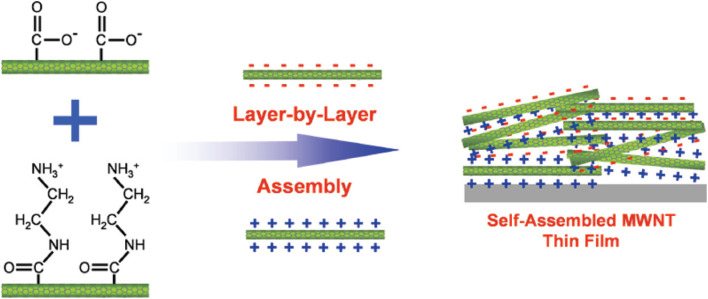
Layer-by-layer assembled MWNT thin film with positively and negatively charged MWNTs. This figure has been adapted/reproduced from ref. 79 with permission from American Chemical Society, copyright 2008.

The LbL assembly on the other hand is a versatile technique for fabricating ultrathin films with precise control over composition and structure at nanoscale. As shown in Fig. 3, the LbL assembly technique involves the sequential deposition of species with opposite charges onto a substrate, typically through electrostatic interactions.^78^ The process generally follows these steps; a substrate is exposed to a solution containing positively charged species. Excess material is washed away. The substrate is then exposed to a solution with negatively charged species. Another washing step removes unbound material. This cycle is repeated to build up multiple layers with nanometer-scale precision. While electrostatic interactions are most common, other driving forces like hydrogen bonding can also be utilized.^1^ This method has proven particularly useful for incorporating CNMs into functional thin films for electrochemical sensing.^79^

The deposition methods significantly influence the charge transport mechanisms in electrochemical sensing by dictating the structure, thickness, and organization of the sensing films.^80,81^ In LbL assembly, the sequential buildup of multilayers allows for the incorporation of conductive CNMs, which facilitate electron transfer and typically result in faradaic processes governed by redox reactions at the electrode interface.^82^ However, if insulating polymers or biolayers dominate the multilayer stack, the system may display non-faradaic behavior due to capacitive effects or hindered charge mobility.^81^ The LB films can support either mechanism depending on the material used; electroactive films promote faradaic currents,^83,84^ while purely insulating layers exhibit non-faradaic, capacitive behavior.^85,86^ LS deposition, by horizontally transferring 2D materials such as graphene sheets onto a substrate, can optimize in-plane charge transport and surface contact, thus often enabling efficient faradaic responses in electrochemical reactions.^87^ These studies collectively illustrate that when CNMs are assembled *via* LbL, LB, or LS, the resulting film morphology and conductivity favor faradaic charge transport, especially when conductive or redox-active materials are incorporated. Non-faradaic (capacitive) responses tend to emerge only when films include insulating layers or lack percolating conductive pathways (ref. 88 and references therein). Hybrid LB/LB films with nanoparticles or conjugated polymers can be achieved for a controlled charge transport and tunable sensor responses.^89^

## 4. Ultrathin films of CNMs and their electrical characteristics

Ultrathin films of CNMs fabricated using LB and LS deposition techniques have several advantages over bulk CNMs for sensor applications. For instance, the thickness, porosity, and surface functionalization of CNMs thin films can be controlled to optimize the sensitivity, selectivity, and stability of the electrochemical sensors.^90,91^ The LB and LS deposition have been used to fabricated ultrathin films of CNMs monolayers onto solid substrates with tailored properties.^75,92,93^ The technique allows for the re-engineering and manipulation of structures on nano- and microscales, leading to ultrathin films with tailored properties, such as high conductivity, and electrocatalytic activity.^94^ The fabrication of ultrathin films of CNMs with LB and LS techniques and their characterization is discussed further in this section.

### 4.1. Fullerenes

Following the discovery of fullerenes, fabrication and characterization of the Langmuir films (LF) of C_60_ presented significant challenges due to the tendency of fullerenes to aggregate into disordered structures, resulting in unpredictable properties. In typical procedures, C_60_ fullerenes were dispersed in benzene and deposited onto a pure water subphase using a microsyringe. These fullerenes formed rigid monolayers with a thickness corresponding to two fullerene molecules. The critical surface pressure at which the fullerene monolayer collapsed was found to be relatively high, approximately π_c_ ∼ 50 mN m^−1^.^95,96^ These films retained discrete π systems and displayed a significant photocurrent response that increased with transfer surface pressure, indicating effective photoinduced charge separation and improved electronic coupling at higher surface densities.

Recent research focuses on LF of functionalized fullerenes, where the incorporation of various functional groups alters the amphiphilicity and reactivity of the fullerenes, resulting in distinct assembly patterns. The LB films of fullerenes are primarily utilized to investigate the fractal growth of polymers at interfaces or the interactions between C_60_ and pyrene-based amphiphiles. Giant amphiphilic molecules such as C_60_-PEO_5_k, C_60_-PEO_5_k-C_60_, C_60_-PEO_10_k, and C_60_-PEO_10_k-C_60_ were spread at the air–water interface to form monolayer films.^97^ Compression of these monolayers increased the packing density of the giant amphiphilic molecules, thereby altering the surface pressure. Subsequently, the monolayer films were transferred onto solid substrates at a specified surface pressure using the LB deposition method. The crystallization ability of polyethylene oxide (PEO) segment induced fractal growth patterns in the LB films. AFM analysis revealed that both the length of the PEO chains and the capping mode had significant effects on the fractal growth and morphology of the LB films.

LB thin films of amphiphilic fullerene derivatives have been synthesized and characterized, highlighting novel fabrication approaches for constructing aligned donor–acceptor arrays through strong intermolecular interactions among fullerene molecules.^95,98,99^ These investigations have focused on the formation of densely packed LMs at air–water interface, which are subsequently transferred onto solid substrates *via* LB deposition, resulting in multilayered thin films exhibiting broadened optical absorption features. The packing density of the monolayers has been reported to be comparable to that of a C_60_ monolayer surface area. Tang *et al.* synthesized an amphiphilic fullerene conjugate bearing oligo (ethylene glycol) and hexadecylaniline units, enabling well-ordered monolayers with significant potential for electronic applications. Their LB films showed organized packing and the potential for π–π stacking, favoring electron mobility through conjugated domains.^100^ Other studies have also reported the successful deposition of mixed fullerene/phthalocyanineato metal monolayers as LB films by the vertical lifting method.^101^ The Langmuir monolayer-forming ability of a C_60_ derivative at the air–water interface was investigated using surface pressure–area (π–A) isotherms and Brewster angle microscopy (BAM).^101^ The resulting monolayers were transferred onto quartz, mica, and ITO substrates *via* LB technique and subsequently characterized by AFM. Cyclic voltammetry revealed that the LB film of the C_60_ derivative exhibits ionic charge-selective electron-transfer behavior, suggesting its potential application as an electrode modifier when functionalized with suitable groups.^95,102^

### 4.2. Single and multi-walled carbon nanotubes

SWCNTs are remarkable nanomaterials with exceptional properties like high electrical conductivity, optical tunability, and mechanical strength. These characteristics make them promising candidates for electrochemical sensing applications.^103–105^ However, their inherent insolubility and tendency to aggregate pose challenges in processing and manipulating them for sensing applications. Poonia *et al.* demonstrated that the alignment of pristine SWCNT bundles can be effectively controlled during the fabrication of ultrathin films using LB technique.^104,106^ Uniform dispersion of SWCNTs is typically achieved by ultrasonication in a dimethylformamide (DMF) solution. The solution is then filtered using filter paper and the LF of SWCNTs was formed by spreading the filtered solution onto the aqueous subphase. The LB deposition technique enables controlled alignment of SWCNTs, either parallel or perpendicular to the dipping direction with respect to patterned interdigitated electrodes (IDEs). The resulting LFs of SWCNTs exhibit notable stability and reversibility at the air–water interface. Surface pressure–area isotherms display well-defined phase transitions, including gas-like, liquid-like, and collapse regions, indicative of an organized monolayer structure. The field emission scanning electron microscopy (FE-SEM) characterization confirms the preferential orientation of SWCNTs along a dominant axis within the film. Electrical characterization *via* current–voltage (*I*–*V*) measurements further substantiates the anisotropic alignment, revealing distinct conductive behavior depending on whether the SWCNTs are aligned parallel or perpendicular to the IDE configuration.^104^

**Fig. 4:**
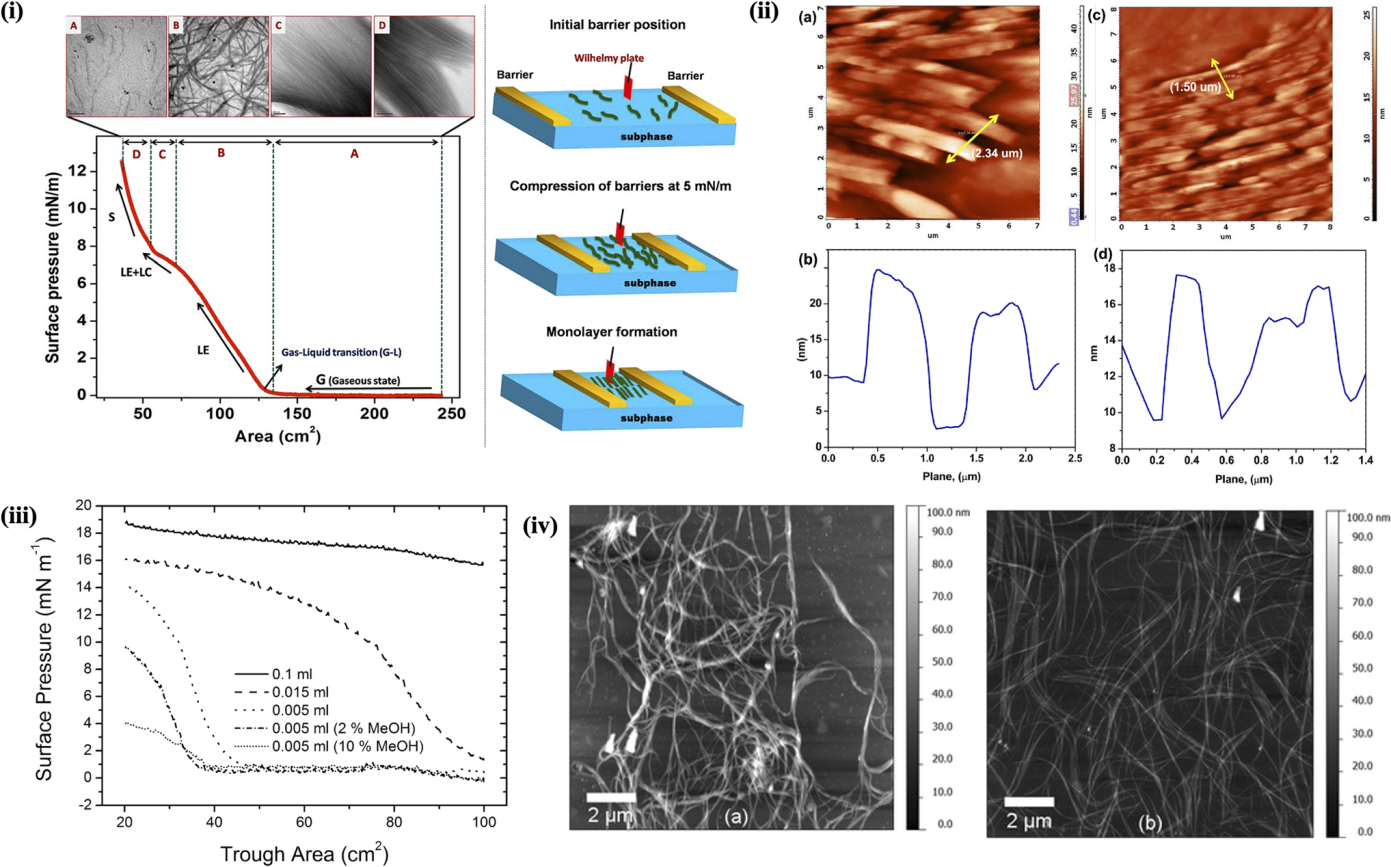
(i) π–A isotherms of PANI@MWCNTs at the air–water interface with corresponding TEM images transferred at each phase (A–D) and a schematic representation of the formation of aligned Langmuir monolayers at the air–water interface as a function of surface pressure. (ii) AFM images of LB films of aligned PANI@MWCNTs transferred at 10 mN m^−1^ onto silicon substrates and corresponding line scan profiles. Figures (i) and (ii) have been adapted/reproduced from ref. 110 with permission from American Chemical Society, copyright 2020. (iii) π–A isotherms for semiconducting carbon nanotubes, showing the influence of addition of methanol to the subphase. (iv) AFM images of (a) a 5-layer metallic SWCNTs LB network and (b) a 5-layer semiconducting SWCNTs LB network. Figures (iii) and (iv) have been adapted/reproduced from ref. 107 with permission from American Chemical Society, copyright 2012.

On the other hand, by exploiting their amphiphilic character, SWCNTs can be dispersed in aqueous solutions containing surfactants or co-solvents. At the air–water interface, these amphiphilic molecules self-assemble into ordered monolayers, with the hydrophobic SWCNTs aligning parallel to the interface and the hydrophilic head groups extending into the water phase.^107,108^ Massey *et al.*^107^ utilized LB deposition technique to form thin film networks of both metallic and semiconducting SWCNTs and investigated their physical, optical, and morphological properties. Fig. 4iii represents π–A isotherms for semiconducting carbon nanotubes, highlighting the effects of both the deposited material quantity on the surface and methanol incorporation into the subphase. The electrical conductivities of LB films have been investigated over a temperature range of 80–350 K and across electrode gaps of 220 nm and 2 mm. The AFM images presented in Fig. 4iv display 5-layer LB films deposited on palladium after annealing at 150 °C for 30 minutes. These images are significantly sharper, clearly revealing distinct bundles of CNTs without any indication of extraneous material. For semiconducting tubes, the findings indicate that Poole–Frenkel conduction is the primary electrical mechanism at temperatures under 150 K and electric fields exceeding 1 MV m^−1^. In contrast, metallic nanotube networks show a reduction in resistance as the temperature decreases, yielding a temperature coefficient of resistance of 10^−3^ K^−1^. Sgobba *et al.*,^11^ demonstrated that SWCNTs can be dispersed in 1,2-dichloroethane (DCE) through noncovalent functionalization using a synthetic polymer (referred to as polymer 1), which wraps around small bundles of nanotubes and disrupts the strong van der Waals forces holding the SWCNTs together. This approach yields a highly stable 1/SWCNT suspension that can be spread at air–water interface using a Langmuir trough. The resulting floating film is then transferred onto solid substrates, and spectroscopic analysis with polarized light indicates the formation of a well-aligned thin film. Photoelectrochemical cells constructed with ITO/(1/SWCNT)_*x*_ photoelectrodes, prepared *via* LS method, display a significantly enhanced photocurrent compared to those made by spin coating. Han *et al.* fabricated a series of composite films combining amino-functionalized multi-walled carbon nanotubes (MWCNTs-NH_2_) with three different dye molecules, including methylene blue, neutral red and safranine T, using LB technique.^109^ Among these, the MWCNTs-NH_2_/methylene blue composite film demonstrated not only the highest photocurrent output but also the lowest impedance compared to the other films, signifying reduced resistance, improved carrier transport, and enhanced stability.

The successful fabrication of well-aligned ultrathin films of polyaniline-functionalized multi-walled carbon nanotubes (PANI@MWCNTs) with high orientational order over macroscopic areas using LB technique has been demonstrated by Abdulla *et al.*^110^ The interfacial assembly of PANI@MWCNTs at the air–water interface results in the gradual formation of locally ordered domains, which coalesce into a well-oriented monolayer (Fig. 4i). The formation and stability of compact monolayer and multilayer structures in PANI@MWCNTs-based LB films have been extensively examined using π–A isotherms and thermodynamic analysis. The orientation and alignment of PANI@MWCNTs within LF at the air–water interface were systematically investigated as a function of interface temperature, employing transmission electron microscopy (TEM) to provide detailed structural insights. AFM images of LB films of aligned PANI@MWCNTs transferred at 10 mN m^−1^ onto silicon substrates and corresponding line scan profiles are shown in Fig. 4ii. Surface functionalization of MWCNTs with PANI has been shown to mitigate the 3D aggregation of CNTs, promoting the formation of an oriented assembly of PANI@MWCNTs. In a separate study, Chen *et al.* developed a modified glassy carbon electrode (GCE) by assembling phenylsulfonic group-grafted MWCNTs with dye molecules using LB technique for the detection of trace cadmium (Cd^2+^) ions *via* square wave anodic stripping voltammetry (SWASV). The synergistic interaction between the MWCNTs and dye molecules, along with the orderly arrangement in the composite LB films, significantly enhanced the sensor's detection performance.^111^

### 4.3. Graphene and graphene oxide

The LB technique has been employed as an efficient method to control the interfacial molecular orientation and packing of graphene sheets, enabling the creation of patterned conductive networks with precise structural control on flexible and stretchable substrates.^23,112–115^ For instance, Devida *et al.*^116^ reported the direct immobilization of glucose oxidase (GOx) onto GO-based electrodes fabricated *via* LB assembly. The oxygen-containing functional groups on GO facilitated the direct attachment of GOx by simply exposing the electrodes to the enzyme solution. By electrochemically reducing GO to rGO, the surface chemistry could be tuned, allowing control over the density of enzyme binding sites and the overall responsiveness of the LB biofilm.

**Fig. 5:**
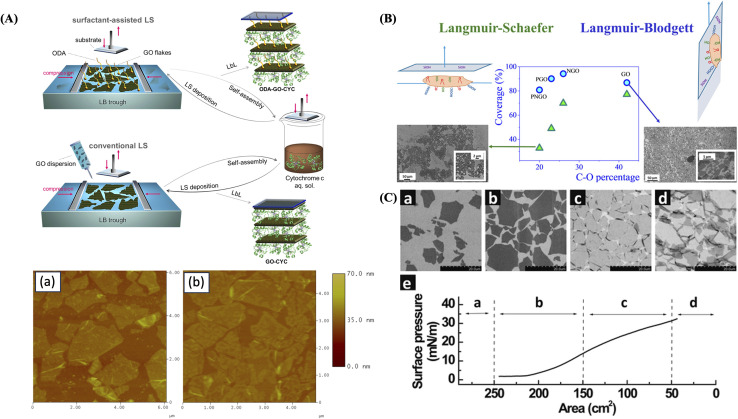
(A) Schematic representation of surfactant-assisted LS and conventional LS deposition of the synthetic procedures. AFM height images of GO–CYC (a) and ODA–GO–CYC (d) multilayer thin films. Figure (A) has been adapted/reproduced from (ref. 117) with permission from American Chemical Society, copyright 2012. (B) Diagram illustrating groups involved in contact between solid substrate (Si/SiO_2_) and GO sheets for LB and LS methodologies. SEM images and coverage percentages for different GO materials for LB and LS methodologies. Figure (B) has been adapted/reproduced from ref. 118 with permission from American Chemical Society, copyright 2015. (C) Langmuir–Blodgett assembly of graphite oxide single layers. (a–d) SEM images showing the collected graphite oxide monolayers on a silicon wafer at different regions of the isotherm. (e) π–A isotherm showing the corresponding regions (a–d) at which the monolayers were collected. Scale bars in (a–d) represent 20 μm. Figure (C) has been adapted/reproduced from ref. 119 with permission from American Chemical Society, copyright 2009.

In another study, Song *et al.*^14^ presented a simple, clean method to create GO–dye composite films using the LB method at room temperature. Interactions between cationic dye molecules and negatively charged GO sheets promote spontaneous film formation through electrostatic and π–π interactions. GO sheets act as a platform for dye self-assembly, resulting in ordered structures with H- and/or J-aggregates. Morphological analysis indicates the formation of smooth and densely packed composite films, whereas spectroscopic characterization confirms the presence of well-organized dye aggregates on GO surfaces. This study offers new insights into preparing organized composite films with potential applications across various fields. Chalmpes *et al.*^117^ fabricated biohybrid graphene-based multilayer architectures utilizing both conventional and amino surfactant-assisted LS deposition techniques (Fig. 5A). In these structures, cytochrome c (CYC) molecules were incorporated between ordered layers of GO. The densely arranged array illustrates controlled formation of GO–CYC hybrid layers (Fig. 5A(a)). The AFM images of the ODA–GO–CYC film (Fig. 5A(b)) reveal flakes with lateral sizes varying from 500 nm to 3 μm. Analytical characterization results demonstrated the biocatalytic activity of these nano-bioarchitectures by testing their performance in the enzymatic oxidation and facilitating the decolorization of pinacyanol chloride. The multilayer structures demonstrated high biocatalytic activity and stability, when surfactant molecules were not used during monolayer deposition. Hidalgo *et al.*^118^ employed both LB and LS methods to fabricate GO thin films (Fig. 5B). Their findings showed that the chemical composition of GO sheets can be tailored by chemically oxidizing either graphite or graphene nanofibers as starting materials. SEM images revealed that the LB technique achieves the highest film coverage (Fig. 5B). In contrast, for films prepared using the LS method, the solid coverage increases with a greater number of C–O groups attached to the basal plane of the GO sheets. These results demonstrate that both the chemical composition of GO and the chosen deposition method can be used to modulate the coverage of GO films. Cote *et al.*^119^ demonstrated that LB assembly enables the formation of water-supported monolayers of graphene oxide single layers (GOSLs) without the need for surfactants or stabilizers. These monolayers remain stable at the air–water interface due to edge-to-edge repulsion, which prevents overlapping during compression. At high surface pressures, the layers wrinkle and fold at the edges while the interiors stay flat (Fig. 5C). The monolayers can be easily transferred to solid substrates with tunable density, ranging from dilute to overpacked arrangements. When GOSLs of different sizes are stacked face-to-face, they form irreversible double layers. The monolayers are readily visualized by SEM (Fig. 5C(a–d)), with clear contrast between single and multiple layers. These findings provide valuable insights into thin-film processing of GO, as the packing of GOSLs influences surface roughness, porosity, and density. Moreover, LB assembly offers a straightforward route to large-area GOSL monolayers, which are promising precursors for graphene-based electronics.

Additionally, a modified Langmuir–Blodgett (MLB) technique has been introduced as a straightforward, economical, and scalable alternative to traditional LB methods for transferring GO monolayers onto solid substrates.^115^ The MLB method utilizes a basic setup comprising a glass or Teflon reservoir and a stationary substrate. GO sheets are transferred by carefully draining the subphase, resulting in uniform, flat, and well-adhered GO layers on RCA-1 treated Si and SiO_2_/Si substrates under various deposition conditions by varying the subphase pH (3.5–6.5), target pressure (1–12 mN m^−1^) and meniscus speed (1–10 mm min^−1^). Chemical reduction of GO sheets deposited by MLB results in rGO that exhibits properties comparable to those achieved through LB and other techniques. This technique offers a versatile approach for creating high-quality GO and rGO sheets suitable for device applications on solid substrates.

### 4.4. Other carbon nanomaterials

**Fig. 6:**
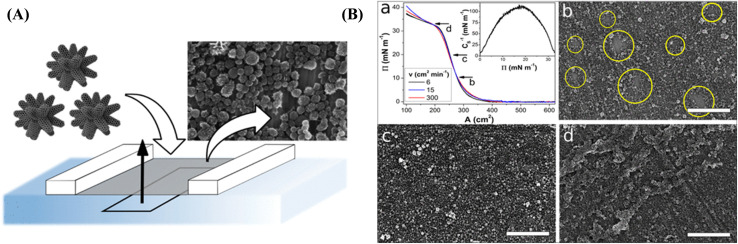
(A) A suspension of single-walled carbon nanohorns (SWCNHs) aggregates with a size of approximate 50 nm was used to create a floating film at the air–water interface. (B) π–A isotherms of SWCNH floating films (a) and the corresponding compressibility module plot (inset). SEM images of SWCNH films transferred at π = 10, 20, and 33 mN m^−1^ (b–d, respectively); scale bars represent 2 μm. These figure have been adapted/reproduced from ref. 76 available under a Creative Commons Attribution 4.0 International License.

Recent research has demonstrated significant progress in the formation of thin films of other CNMs using the LB and LS technique. Kędzierski *et al.*^76^ presented the fabrication and characterization of LS films composed of single-wall carbon nanohorns (SWCNHs). By adjusting the surface pressure during layer transfer, the density and arrangement of SWCNHs within the film are precisely regulated (Fig. 6A). These nanohorns films exhibit transparency levels comparable to other carbon nanostructure thin films. However, their resistivity surpasses that of carbon nanotube thin films, aligning more closely with defective graphene nanoflake layers. While SWCNH films share similar optical and electrical properties with other nanocarbons, they possess several distinct advantages. Unlike CNTs, SWCNHs are free from metal ion contamination often introduced during production. SWCNHs boast a significantly larger specific surface area due to their tendency to form “dahlia-like” aggregates (Fig. 6B). These unique attributes position carbon nanohorns as highly promising candidates for two key applications as model compounds for investigating complex intermolecular interactions as scaffolds for developing advanced photoelectrochemical sensors. The combination of these properties and potential applications underscores the significance of SWCNH-based LS films in the field of nanomaterials and sensor technology. In another study, kinetics of hydrophobic carbon quantum dots (hCQDs) have been investigated, providing insights into the potential applications of these films in electrochemical sensing.^22^ This study investigates the formation of Langmuir films using hCQDs. Bodik *et al.* optimized the pyrolysis kinetics of hCQDs to maximize their photoluminescence and characterized the resulting nanoparticles using TEM, X-ray photoemission spectroscopy (XPS), and grazing-incidence X-ray diffraction. The monolayer formation process was monitored in real-time during continuous compression, measuring surface pressure, surface potential, elastic modulus, and employing BAM.

Carbon can adopt sp, sp^2^, and sp^3^ hybridization states, each leading to the formation of nanomaterials with distinct structural and electronic properties that are highly relevant to electrochemical sensing. Recent reviews describe how the electronic hybridization of carbon, specifically the distinction between sp^2^ and sp^3^ configurations, fundamentally shapes the performance of carbon-based ECSs.^120,121^ For instance, sp^2^-hybridized nanocarbons such as graphene and CNTs possess delocalized π-electron systems that confer exceptional electrical conductivity, facilitate rapid electron-transfer kinetics, and offer abundant functionalization sites, all critical for achieving high sensitivity and fast sensor response in biosensing and gas detection applications.^122,123^ In contrast, sp^3^-hybridized materials like BDD exhibit a wide electrochemical potential window (typically >3 V in aqueous media), extremely low background current, and outstanding chemical and mechanical stability, making them particularly suited for trace-level detection in demanding or fouling-prone environments.^67^ Thus, selecting between sp^2^ or sp^3^ carbon frameworks enables deliberate tailoring of sensor attributes. Meanwhile, sp-hybridized carbon, as found in carbyne or polyynes, exhibits promising linear π-conjugated one-dimensional structures that theoretically offer highly efficient charge transport; however, due to stability and fabrication challenges, its direct application in electrochemical sensing remains largely conceptual and at an early research stage.^124,125^

## 5. Applications of ultrathin films of CNMs for high performance ECSs

The ultrathin films of CNMs have been utilized in various ECSs, including gas sensing, biosensing, and environmental monitoring.^2,103,104^ For electrochemical applications, the electrochemical behavior of CNMs monolayers can be assessed using techniques such as electrochemical impedance spectroscopy (EIS), chronoamperometry, CV, DPV, and square wave voltammetry (SWV). These techniques offer insights into the electrical characteristics and efficacy of single-layer materials when they interact with target substances in electrochemical detection systems. These approaches help elucidate how the monolayers respond electrically and perform their sensing functions when exposed to specific analytes in electrochemical sensing applications. The advanced ECSs that utilize ultrathin films of CNMs are discussed further in section. These high-efficiency sensing devices, which find applications across diverse sectors, are examined in greater detail. The focus is on how these CNM-based ultrathin films contribute to the development of cutting-edge ECSs and their implementation in various fields of study and industry.

### 5.1. Gas sensors

CNMs are highly promising for electrochemical gas sensing due to their exceptional properties. Their large surface area offers abundant active sites for gas interaction, while excellent electrical conductivity enables efficient electron transfer. Coupled with strong chemical stability, these features make CNMs ideal for developing sensitive, selective, and durable gas sensors.^126,127^ Hence, these materials, particularly CNTs and graphene, have been extensively studied for their potential to enhance gas sensor performance. As discussed in previous section, the LB technique enables precise control over the organization and deposition of CNMs monolayers onto sensor surfaces, resulting in ultrathin films with tailored molecular arrangements that enhance the interaction between the sensing interface and target gases.^128^ LB-fabricated CNMs films have been successfully used to detect various gases, including ammonia,^110^ methane,^104^ carbon dioxide (CO_2_),^129^ acetone^130^ and hydrogen.^131^

Andrić *et al.*^129^ presented a CO_2_ gas sensor fabricated from LB thin films of sulfonated polyaniline-functionalized graphene, produced by electrochemical exfoliation. This approach leverages the unique properties of functionalized graphene and the precise molecular organization enabled by the LB technique to achieve sensitive CO_2_ detection. It demonstrated high sensitivity and a wide dynamic range, responding in about 15 seconds. The sensor maintained stable performance over several months and was used for real-time CO_2_ monitoring. A rapid-response hydrogen gas sensor was fabricated using LB film of palladium-decorated single-walled carbon nanotubes (Pd-SWCNTs).^131^ Integrated onto a gold interdigitated transducer, the sensor demonstrated sensitive and reversible detection of hydrogen concentrations ranging from 0.025% to 2.5% (v/v) in nitrogen at room temperature. Optimal performance was achieved with 10 μM palladium acetate concentration. Higher concentrations altered film conductivity, reducing sensor effectiveness. This Pd-SWCNT sensor design offers potential for detecting various gaseous molecules. In another study, Kumar *et al.*^130^ demonstrated ultrathin LB films of octadecylamine-functionalized single-walled carbon nanotubes (ODA-CNTs) for acetone vapor sensing film. Impedance spectroscopy measurements at room temperature revealed that ultrathin films of ODA-functionalized carbon nanotubes (ODA-CNTs) outperformed both drop-cast ODA-CNTs and LB films of unmodified CNTs. The alignment of nanotubes in the LB films enabled a low detection limit of 0.5 ppm and a broad sensing range of 1–300 ppm for acetone. Principal component analysis (PCA) indicated that capacitance is the most effective parameter for acetone detection. These findings highlight the potential of LB films of ODA-CNTs for highly sensitive, low-level acetone sensing at room temperature, with promising applications in industrial effluent monitoring and as a non-invasive biomarker for diabetes.

**Fig. 7:**
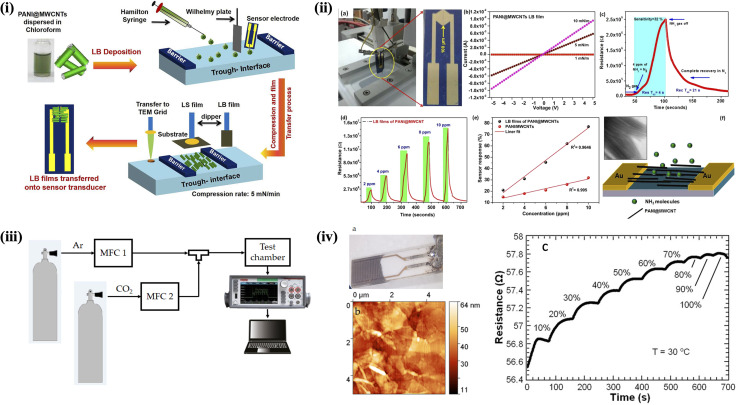
(i) Schematic (left) of LB assembly of PANI@MWCNTs onto the sensor electrodes. (ii(a)) Vertical dipping of PANI@MWCNT LB films on Au double electrodes. (b) *I*–*V* characteristics at various pressures. (c) Single NH_3_ gas sensing profile for 4 ppm. (d) Dynamic NH_3_ gas sensor response of LB films of PANI@MWCNTs. (e) Sensitivity plots of spin-coated films and LB films of the PANI@MWCNTs. (f) Schematic representation of the gas sensing process on aligned PANI@MWCNT LB films. Figures (i) and (ii) have been adapted/reproduced from ref. 110 with permission from American Chemical Society, copyright 2020. (iii) Schematic of the experimental setup. (iv) Sample characterization: (a) optical photograph of the graphene sensor; (b) atomic force micrograph of the sensor surface. (c) Sensor response to CO_2_ across the full volume concentration range of 0% to 100%. Figures (iii) and (iv) have been adapted/reproduced from ref. 129 available under a Creative Commons Attribution 4.0 International License.

Abdulla *et al.*^110^ demonstrated the highly ordered, ultrathin films composed of PANI@MWCNTs using the LB technique (Fig. 7i). The PANI@MWCNTs self-assemble into oriented monolayers at the air–water interface, with their alignment behavior studied across various temperatures, including 10 °C, 15 °C, 20 °C, 25 °C, 30 °C. The LB films were transferred onto the sensor electrodes by a vertical dipping method at 10 mN m^−1^ (Fig. 7ii(a)). The PANI functionalization effectively prevents 3D aggregation of CNTs, promoting directional assembly. The resulting LB films exhibit enhanced ammonia gas sensing properties at room temperature compared to randomly oriented networks, attributed to the directed electron transport facilitated by the alignment of PANI@MWCNTs. Fig. 7ii(b) presents the *I*–*V* characteristics of the transferred PANI@MWCNT monolayer. This large-scale alignment enables the scalable fabrication of high-density, MEMS-integrated nanosensor arrays, offering improved sensitivity and reliability for NH_3_ detection. Fig. 7ii(c) presents the response and recovery times of sensors based on LB films of PANI@MWCNTs when exposed to 4 ppm of NH_3_ gas at room temperature. The dynamic response of the sensor to trace concentrations of NH_3_ is illustrated in Fig. 7ii(d). Andrić *et al.*^129^ reported the fabrication of CO_2_ gas sensors using LB thin films composed of sulfonated polyaniline-functionalized graphene, where the graphene was produced *via* electrochemical exfoliation. The sensor was placed in a custom gas sensing chamber alongside a Pt1000 thermometer (Fig. 7iii). Gas flow was controlled at 190 mL min^−1^ using mass flow controllers. After purging with argon, precise CO_2_ concentrations were introduced using calibrated gas cylinders and further diluted with argon as needed. Sensor resistance was monitored in real time with a digital multimeter as CO_2_ concentrations varied. A photograph of the graphene sensor is shown in Fig. 7iv(a). Fig. 7iv(b) represents an atomic force micrograph of the sensing surface. The sensor's active layer consists of graphene flakes distributed across the substrate's surface. Fig. 7iv(c) shows the measured two-terminal resistance of the graphene sensor as the CO_2_ volume concentration increases from 0% to 100% at atmospheric pressure. As the CO_2_ concentration rises, the resistance increases from an initial value of 56.5 Ω to 57.8 Ω. Additionally, the LB technique has been effectively employed to tailor the surface chemistry ofGO, further enhancing its performance in gas sensing applications.^132^ Furthermore, LB films of CNMs have been used in alcohol detection^133^ and in the development of toxic gas sensors.^134,135^Table 1 summarizes the different sensor types, including the deposition method, film thickness, limit of detection (LOD), and analytes detected.

**Table 1:** Summary of CNM based ECSs: deposition techniques, materials, and analytes detected

Sensor type	Deposition technique	CNMs composite	Film thickness	Analyte detected	LOD	Analytical technique	Reference
Gas sensors	Langmuir–Blodgett	PANI@MWCNTs	8–10 nm	NH_3_	2 ppm	Transducer, changes in electrical resistance	Abdulla *et al.* (2020)^110^
	Langmuir–Blodgett	SWCNTs	30 nm	Methane	10 ppm	Kelvin probe measurement	Poonia *et al.* (2015)^104^
	Langmuir–Blodgett	Pd-SWNT	1 LB monolayer	Hydrogen	0.025–2.5% (v/v)	Transducer, changes in electrical resistance	Lee *et al.* (2013)^131^
	Langmuir–Blodgett	PANI@graphene	11.5 nm	CO_2_	150 ppm	Changes in electrical resistance	Andrić *et al.* (2021)^129^
	Langmuir–Blodgett	ODA-CNT	1 LB monolayer	Acetone	0.5 ppm	Impedance analyzer	Kumar *et al.* (2024)^130^
	Langmuir–Blodgett	SWCNTs-onto-CdA (cadmium arachidate)	2 LB monolayers	VOCs	N/A	QCM, SAW, silica optical fiber (SOF)	Penza *et al.* (2005)^148^
	Langmuir–Blodgett	SWCNTs-onto-CdA	56 nm	Alcohol	N/A	QCM and SOF	Penza *et al.* (2004)^133^
Bio sensors	Langmuir–Blodgett	Nafion-nitrogen doped carbon nanotubes	1 LB monolayer	Caffeine	≈3.88 ppb	SWASVs	Wu *et al.* (2019)^138^
	Langmuir–Blodgett	MWCNTs-LB/GCE	1 LB monolayer	Methylparaben	15.2 ppb	Chronocoulometry	Wang *et al.* (2015)^139^
	Layer-by-layer	AuNPs-PAH/CNTS	10 bilayers	Cholesterol	5.72 ppm	Amperometric	Silva *et al.* (2019)^137^
	Langmuir–Schaefer	ODA–GO–CYC	1.4 ± 0.3 nm	Cytochrome c as biocatalytic activity indicator	N/A	Oxidation, changes in absorbance	Chalmpes *et al.* (2022)^117^
	Langmuir–Blodgett	ODACNTs onto glucose oxidase	1 LB monolayer	Glucose	10 pM	Cyclic voltammetry	Gayakwad *et al.* (2023)^142^
	Langmuir–Blodgett	DPPE immobilized GOx	5–15 nm	Glucose	0–0.9 mg mL^−1^	Acoustoelectronic	Gorbachev *et al.* (2023)^136^
	Electrodeposition	AgNPs/rGO/GCE	NA	H_2_O_2_	3.19 μM	Cyclic voltammetry	Zhang *et al.* (2024)^164^
	Electrodeposition	AgNPs/rGO/GCE	NA	Dopamine	0.18 μM	Cyclic voltammetry	Zhang *et al.* (2024)^164^
	Langmuir–Blodgett	Carboxylated graphene	1 LB monolayer	Urea	≈5 ppm	QCM	Poonia *et al.* (2018)^45^
	Langmuir–Blodgett	Densely aligned CNT array	≈1.5 nm	AD biomarker	2.13 to 2.72 fM	Multiplexed electrical sensing	Kim *et al.* (2020)^140^
	Langmuir–Blodgett	DMPA-CNTs-PEN films	1 LB monolayer	Penicillin G	10 μM	EIS	Scholl *et al.* (2017)^141^
Environmental sensors	Layer-by-layer	gPBAT(PPY/CNT)_*n*_	20 bilayers	Paraquat herbicide	0.073 μM	Cyclic voltammetry and EIS	Amaro *et al.* (2023)^152^
	Layer-by-layer	PDDA/rGO	5 bilayers	Carbofuran pesticide	0.407 μmol L^−1^	DPV	Miyazaki *et al.* (2020)^153^
	Layer-by-layer	MWCNT/NiTsPc	10 bilayers	Diquat herbicide	9.62 × 10^−7^ mol L^−1^	DPV	Zattim Jr *et al.* (2024)^165^
	Langmuir–Schaefer	ODA-SWCNTs	1 LB monolayer	Fluoride ion	0.5 ppm	eQCM and cyclic voltammetry	Taneja *et al.* (2023)^146^
	Langmuir–Blodgett	MWCNT-SO_3_H	2 LB monolayer	Cd(ii) ion	0.08 μM	SWASV	Chen *et al.* (2018)^111^
	Langmuir–Blodgett	PANI@MWCNTs	Multilayers	Daidzein	8 × 10^−8^ mol L^−1^	Chronocoulometry, cyclic voltammetry, LSV, EIS	Wang L., *et al.* (2016)^144^
	Exfoliated with an ultrasound tip	GO–BiNPs nanocomposite	N/A	Pb(ii)	30 nmol L^−1^	Anodic stripping under square wave voltammetry (ASSWV), CV	Bindewald E. H., *et al.* (2017)^145^
	Exfoliated with an ultrasound tip	GO–BiNPs nanocomposite	N/A	Cd(ii) ion	27 nmol L^−1^	ASSWV, CV	Bindewald E. H., *et al.* (2017)^145^
Wearable ECSs	Wet spinning/fiber formation	CNT fibers	N/A	Glucose, dopamine	N/A	Amperometry, cyclic voltammetry	Lee *et al.* (2020)^156^
	Vacuum filtration & film transfer	CNTs	Few μm	Lactate	mM range	Biofuel cell-based amperometry	Chen *et al.* (2019)^157^
	Drop-casting	MWCNT	N/A	Cortisol (sweat)	0.1–10 ng mL^−1^	EIS, amperometry	Tian *et al.* (2023)^158^
	Screen printing	Carbon-based ink	Few μm	Salivary ketone bodies	0.1–10 mM	Voltammetry	Moonla *et al.* (2023)^159^
	Screen printing + drop casting	Carbon black	N/A	Sodium ions in sweat	1–100 mM	Ion-selective potentiometry	Mazzaracchio *et al.* (2021)^161^

### 5.2. Biosensors

As discussed in Section 4, LB and LS techniques are powerful methodologies for fabricating thin films of CNMs, such as graphene and CNTs, for use in electrochemical sensing. These techniques offer several advantages that make it particularly suitable for biosensing applications, including controlled film organization, ease of enzyme immobilization, biomimetic structures, and tailored interfacial characteristics.^91,103,135–137^ Applications of LB-fabricated CNM films in electrochemical biosensors are wide-ranging. For instance, they have been used in the detection of caffeine in tea,^138^ in glucose sensors,^136^ and in biosensors for milk analysis.^91^ A novel voltametric sensor using LB film of MWCNTs on GCE was developed for methylparaben detection.^139^ The MWCNTs-LB/GCE sensor demonstrated high sensitivity, with a linear range of 1 × 10^−6^ to 8 × 10^−5^ mol L^−1^ and a detection limit of 4 × 10^−7^ mol L^−1^. The electrochemical properties of the sensor and the reaction mechanism of methylparaben were studied, with the method successfully applied for analyzing cosmetic samples.

**Fig. 8:**
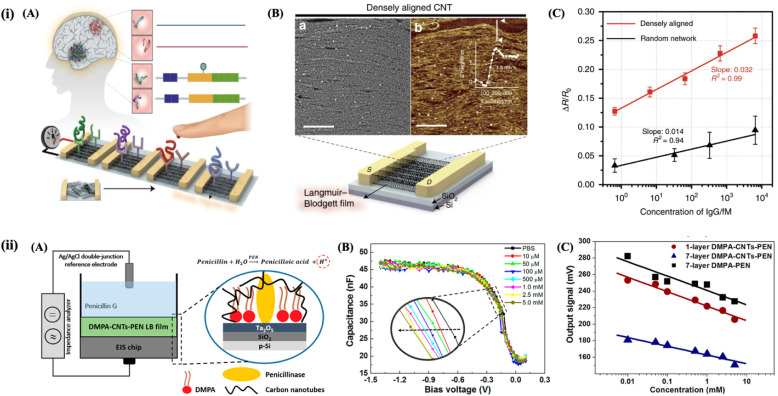
(i(A)) Schematic illustration of a densely aligned CNT sensor array for AD biomarkers. (i(B)) (a) SEM and (b) AFM images of densely aligned LB film of CNT. The scale bars in SEM and AFM images indicate 250 nm and 500 nm, respectively. (i(C)) Sensing performance comparison between aligned and random-network CNT devices. The figure (i) has been adapted/reproduced from ref. 140 available under a Creative Commons Attribution 4.0 International License. (ii(A)) Schematic representation of a capacitive EIS sensor modified with DMPA-CNTs-PEN LB film, and biosensing mechanism. (ii(B)) Capacitance–voltage curves toward penicillin concentrations ranging from 10 μM to 5.0 mM for a 1-layer EIS-(DMPA-CNTs-PEN) sensor. (ii(C)) Calibration curve responses for the LB films correlated with penicillin concentrations. Figure (ii) has been adapted/reproduced from ref. 141 with permission from American Chemical Society, copyright 2017.

Kim *et al.*^140^ prepared densely aligned CNT films at air–water interface using the LB methodology, followed by their transfer onto a silicon substrate (Fig. 8i). A highly accurate and sensitive approach for detecting key Alzheimer's disease (AD) biomarkers in human plasma was developed. These CNT sensor arrays exhibited outstanding precision, sensitivity, and accuracy, characterized by a low coefficient of variation, femtomolar-level detection limits, and high recovery rates. The sensor array analyzes biomarker ratios in clinical blood samples to distinguish AD patients from healthy controls, achieving 90.0% sensitivity, 90.0% selectivity, and 88.6% average accuracy. Gayakwad *et al.*^142^ demonstrated enhanced glucose sensing using a nano-stratified layer of SWCNTs on an electrochemical platform. GOx was immobilized on a highly organized LB film of octadecylamine-functionalized CNTs on a gold electrode surface substrate. The LB film-modified electrode outperformed drop-casted electrodes, offering a wider concentration range (10 pM to 1 mM), lower detection limit (10 pM), and 2.8× better sensitivity. This improved performance is attributed to the organized nanotube structure in the LB film. Scholl *et al.*^141^ demonstrated the integration of CNTs into penicillinase-phospholipid Langmuir and LB films to enhance the catalytic properties of the enzyme penicillinase for biosensing applications (Fig. 8ii). The adsorption behavior of penicillinase and CNTs at dimyristoylphosphatidic acid (DMPA) monolayers at the air–water interface was investigated, and the mixed DMPA-CNTs-PEN films were characterized using various spectroscopic and microscopic techniques. These films were effectively tested as penicillin sensors in a capacitive electrolyte–insulator–semiconductor (EIS) device, showing consistent output signals across all tested concentrations. This study demonstrates the potential of using LB films that combine CNTs, lipids, and enzymes in EIS devices for biosensing applications.

The LB technique has also been used to optimize the surface chemistry of GO for improved performance in biosensing applications.^116^ Furthermore, LB films of CNMs have been used in the development of conjugated polymer-based health biosensors.^143^ In summary, the LB and LS techniques offer a controlled and precise method for fabricating thin films of CNMs for use in electrochemical biosensors. The resulting sensors exhibit enhanced performance characteristics, including improved sensitivity and selectivity, making them highly effective for detecting wide ranged chemical and biological analytes.^35,144^

### 5.3. Environmental monitoring

In environmental monitoring applications, LB-fabricated CNM films have been used to detect various analytes, including heavy metal ions,^111,145,146^ volatile organic compounds,^147,148^ and other environmental pollutants.^45,149–151^ Bindewald *et al.*^145^ presented a novel method for preparing a GO and bismuth nanoparticle (BiNP) nanocomposite for electrochemical response for Cd(ii) and Pb(ii) ions. BiNPs were synthesized using ultrasonic irradiation, with Bi(NO_3_)_3_ serving as the metal precursor and ascorbic acid as the reducing agent. These BiNPs were subsequently used to modify GCEs, resulting in significantly improved electrochemical sensitivity for detecting Pb(ii) and Cd(ii) ions. The modified electrodes displayed a linear detection range of 0.1 to 1.4 μmol L^−1^ for both metal ions, with detection limits of 30 and 27 nmol L^−1^, respectively. Results indicated the nanocomposite's potential for enhancing voltametric procedure sensitivity. A voltametric sensor for trace cadmium ion detection was fabricated *via* a facile self-assembled method, utilizing GCE modified with phenylsulfonic acid-functionalized multi-walled carbon nanotubes (CNT-SO_3_H) and dye molecules *via* LB assembly.^111^ The synergistic effect of the components enhanced detection performance. Under optimized conditions, the sensor exhibited a linear response to Cd^2+^ concentrations in the range of 0.1 to 1.2 μM, achieving a detection limit of 0.08 μM. This simple, non-toxic preparation method resulted in a highly sensitive sensor, highlighting its potential for heavy metal ion detection and environmental analysis.

**Fig. 9:**
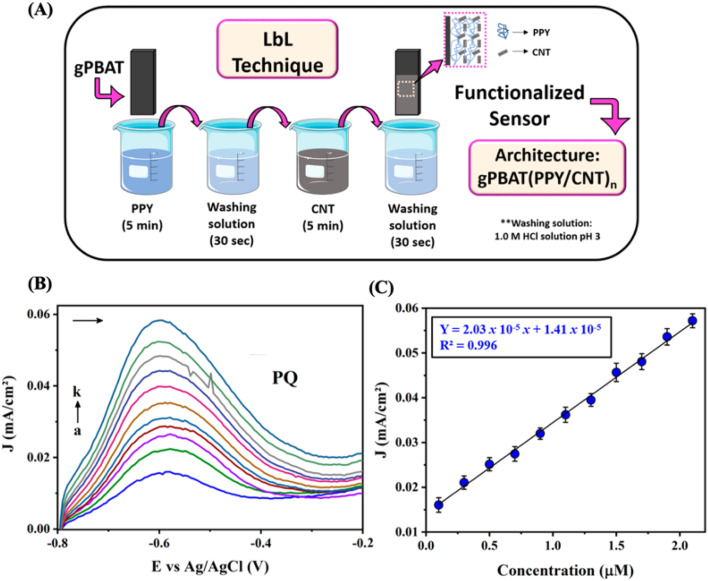
(A) Outline of the gPBAT electrode modification procedure using LbL film gPBAT(PPY/CNT)_*n*_. (B) Electrochemical response for PQ sensor obtained with DPV curves using a concentration ranging from 0.1 to 2.1 μM (a–k). (C) The calibration curve obtained for PQ detection is based on the electrochemical response according to DPV measurements. This figure has been adapted/reproduced from ref. 152 available under a Creative Commons Attribution 4.0 International License.

Amaro *et al.*^152^ investigated flexible sensors constructed from the biodegradable polymer polybutylene adipate-*co*-terephthalate (PBAT) and graphite, utilizing the LbL assembly technique to enhance paraquat detection. The nanostructured films were fabricated by alternately depositing layers of CNTs and polypyrrole (Fig. 9A). Sensors were characterized using various techniques, including SEM, FTIR, CV, and EIS. The detection of paraquat was performed using DPV in a range of 0.1 to 2.1 μM (Fig. 9B), with a detection limit of 0.073 μM. The LbL-modified sensor displayed excellent stability and high recovery rates in tap water samples. These flexible, disposable, and low-cost electrodes show promise for portable environmental analysis. Carbofuran (CBF), a banned pesticide with neurological and carcinogenic risks, persists in global food samples. Miyazaki *et al.*^153^ developed a rapid, low-cost electrochemical sensor for CBF detection using modified ITO electrodes. The two-step LbL modification process involved PDDA/GO and magnetite nanoparticles/PSS films. The sensor, characterized by microscopy and spectroscopy, detected CBF in standard solutions *via* DPV with a sensitivity of 0.2543 (μA cm^−2^ ) (μmol L^−1^) and LOD of 0.407 μmol L^−1^. The sensor was also tested on tap water and soil samples.

LB assembly was employed to fabricate hybrid hetero-structured thin-film electrodes composed of triruthenium clusters integrated with graphene. These electrodes exhibited high efficiency and superior electrochemical performance under high-rate conditions, demonstrating strong potential for supercapacitor applications.^23,109^ These examples illustrate the potential of thin films of CNMs fabricated *via* the LB, LS and LbL techniques for electrochemical environmental monitoring applications. The precise organization and controlled deposition of these films contribute to their enhanced sensitivity and selectivity, making them valuable for detection of environmental pollutants and analytes. As this technology continues to advance, LB graphene-based films have the potential to significantly enhance efficiency and practicality of algal biophotovoltaic fuel cells for sustainable energy production.^75^

### 5.4. Wearable and portable ECSs

The development of wearable and portable ECSs has been significantly advanced by the integration of ultrathin films of CNMs.^89,154,155^ As described in Section 4, ultrathin films of CNMs offer exceptional surface area, conductivity, and mechanical flexibility which are critical properties for on skin or flexible substrates. Combining conducting polymers and CNMs enables flexible, biocompatible, and high-performing wearable/implantable sensors. Their ultrathin morphology enables conformal contact with soft or curved surfaces, facilitating real-time, on-body monitoring of analytes like glucose,^156^ lactate,^157^ cortisol,^158^ and electrolytes in saliva.^159,160^ These sensors can integrate with enzymatic components and ion-selective membranes, enabling multiplexed detection of various biomarkers in sweat or interstitial fluid.^161^ Real-time electrochemical sensing of physiological markers can be achieved through both faradaic electron transfer reactions and non-faradaic impedance changes, depending on device architecture.^89^ Bandodkar *et al.*,^162^ presented highly stretchable, fully printed ECSs and biofuel cells made from a CNT ink combined with an elastomeric binder. These printed devices maintain sensitive and stable electrochemical responses even under significant mechanical deformations, making them a robust platform for wearable and flexible electrochemical sensing. These devices operate without the need for an internal reference solution, enabling miniaturization and integration into portable or wearable platforms.

These advances highlight how ultrathin carbon films serve as the electrochemical interface in miniaturized, low-power systems tailored for personalized health monitoring, sports performance tracking, and remote diagnostics. To achieve high efficiency, further efforts are needed to enhance large-scale production with consistent uniformity and defect-free deposition across various substrates, ensuring reliable performance and high manufacturing yield.^163^

## 6. Computational design for sensor interfaces

The computational landscape for CNMs as smart interfaces in ECSs has rapidly advanced, driven by the integration of multiscale modeling, atomistic simulations, and, more recently, machine learning (ML) and deep learning (DL) methodologies. Early computational work relied on atomistic approaches such as molecular dynamics (MD) and density functional theory (DFT) to elucidate the fundamental electronic, mechanical, and interfacial properties of CNMs, including CNTs and graphene, and to predict their behavior in sensor environments. For example, DFT calculations can predict how strain or chemical functionalization alters the electronic properties of CNTs, directly impacting their sensing performance.^166,167^ MD simulations model the dynamic behavior of atoms and molecules in CNMs, providing insights into structural stability, mechanical properties, and interactions with analytes or electrode surfaces. These simulations are crucial for understanding processes like adsorption, diffusion, and conformational changes at the nanoscale. There are a few other analytical and semi-empirical models which provide detailed, physics-based insights^168–170^ but are beyond the scope of current review. An ideal ECS must possess high selectivity, high sensitivity, and low detection limit, as well as high reproducibility and repeatability. To achieve this, recent studies incorporating both experimental and computational aspects^171–173^ in designing the sensors. For instance, a graphene based ECS was developed for detecting per- and polyfluoroalkyl substances (PFASs) and *ab initio* MD simulations were performed to understand interaction of PFASs with the surface of graphene-based electrode.^172^ In another study, the interaction between acetaminophen (ACOP) and polyethylenimine-functionalized multi-walled carbon nanotubes (PEI-MWCNT) was analyzed using Monte Carlo simulations.^174^

The recent computational advancements particularly the integration of ML and DL methods have significantly transformed the landscape of CNMs as smart interfaces in ultrathin films for high-performance ECSs.^175,176^ For example, ML models can predict how variations in synthesis conditions affect the morphology,^177^ defect density,^178^ functionalization of CNTs and graphene,^179^ and synthesizing CQDs,^180^ thus guiding the rational design of nanomaterial interfaces for enhanced sensor performance. Long Bian *et al.*^181^ combined CNT-based sensing device characteristics with ML to classify and quantify various purine compounds. DL-assisted fluorescence sensor arrays have demonstrated automated, accurate identification of toxic heavy metals in complex matrices, highlighting the potential for on-site, real-time environmental monitoring.^182^ These studies highlight how computational research and ML integration support experimental design, providing guidance on material selection, interface engineering, and optimization of sensors. ML models are increasingly used to process and interpret the complex relationships in experimental data generated by electrochemical sensors and have been reported many ML algorithms or models.^175,176,183^ Particularly, utilizing supervised machine learning models trained on extensive datasets generated by electrical and electrochemical biosensors has become a significant trend in the literature, enabling precise analyses. These computational strategies are not only enhancing the predictive power and efficiency of CNM-based sensor design but are also enabling adaptive, intelligent sensing systems capable of learning from new data and environmental changes. Additionally, they emphasize that modeling is essential for guiding the design and optimization of CNMs and their sensing devices, and that this area is rapidly advancing with new methodologies and computational tools.

## 7. Conclusions and perspective

In conclusion, nano-engineered thin films of carbon nanomaterials (CNMs) have demonstrated remarkable potential in advancing electrochemical sensor (ECS) technology. The unique properties of CNMs, including high electrical conductivity and biocompatibility, coupled with innovative deposition techniques such as Layer-by-Layer (LbL) assembly, Langmuir–Blodgett (LB), and Langmuir–Schaefer (LS), have paved the way for the development of high-performance sensing systems. These sensors exhibit superior analytical capabilities across a wide range of applications, from detecting chemicals and pharmaceutical compounds to identifying biological analytes, environmental pollutants, and gases. In this review we highlighted the significant progress made in utilizing various carbon materials for electrochemical sensing, showcasing their exceptional electrochemical properties and versatility. It also acknowledges the challenges that remain in large-scale implementation and the ongoing search for optimal carbon materials and deposition techniques to further enhance sensor performance. Despite offering exceptional control over film thickness, composition, and molecular ordering, LbL assembly, LB, and LS methods face notable limitations in the context of high-performance electrochemical sensors. These include low scalability, time-consuming fabrication, and material compatibility constraints. While ideal for proof-of-concept studies and fundamental investigations, these techniques are less suited for large-scale sensor production compared to more scalable methods such as spray coating, inkjet printing, or vacuum filtration. Future advances may lie in hybrid approaches that combine the structural precision of LB/LbL techniques with the practicality and speed of scalable deposition methods. The field of nano-engineered CNM thin films for electrochemical sensing is rapidly evolving, with continuous improvements in sensitivity, selectivity, and overall performance.

Looking ahead, the future of CNM-based electrochemical sensors appears promising, with potential for even greater advancements. However, several key areas require further research and development. These include improving the stability and reproducibility of thin films, enhancing the selectivity of sensing materials, and addressing scalability issues for commercial applications. As research progresses, it is anticipated that these challenges will be overcome, leading to the next generation of highly sensitive, selective, and reliable ECSs based on nano-engineered CNM thin films. Such advancements could revolutionize various fields, including healthcare diagnostics, industrial process control, and environmental monitoring, ultimately contributing to improved quality of life and environmental sustainability.

## Conflicts of interest

There are no conflicts to declare.

## Data Availability

No primary research results, software or code have been included, and no new data were generated or analyzed as part of this review.
